# Body Composition Changes and Factors Influencing the Total Weight Loss Rate After Laparoscopic Sleeve Gastrectomy

**DOI:** 10.3390/clinpract14060206

**Published:** 2024-12-05

**Authors:** Hironobu Nakaguchi, Bunzo Matsuura, Teruki Miyake, Hidenori Senba, Shinya Furukawa, Motohira Yoshida, Shigehiro Koga, Yuji Watanabe, Taro Oshikiri, Kumiko Toshimitsu, Yoichi Hiasa

**Affiliations:** 1 Department of Lifestyle-Related Medicine and Endocrinology, Ehime University Graduate School of Medicine, Toon 791-0295, Japan; nakaguchi.hironobu.zf@ehime-u.ac.jp; 2 Department of Gastroenterology and Metabology, Ehime University Graduate School of Medicine, Toon 791-0295, Japan; trk_719@m.ehime-u.ac.jp (T.M.); h.senba0419@gmail.com (H.S.); hiasa@m.ehime-u.ac.jp (Y.H.); 3 Health Services Center, Ehime University, Toon 790-8577, Japan; shinfuru@m.ehime-u.ac.jp; 4 Department of Gastrointestinal Surgery and Surgical Oncology, Ehime University Graduate School of Medicine, Toon 791-0295, Japan; myoshida@m.ehime-u.ac.jp (M.Y.); koga.shigehiro.mg@ehime-u.ac.jp (S.K.); watanabe-y@saijo-c-hospital.jp (Y.W.); oshikiri.taro.th@ehime-u.ac.jp (T.O.); 5 Nutrition Division, Ehime University Hospital, Toon 791-0295, Japan; kumikot@m.ehime-u.ac.jp

**Keywords:** bariatric surgery, percentage of total weight loss, skeletal muscle mass, fat mass

## Abstract

**Objectives:** While the effectiveness of metabolic/bariatric surgery has been confirmed, understanding the factors associated with weight loss is paramount for providing guidance in postoperative treatment strategies. Here, we aimed to examine the factors associated with long-term maintenance of weight loss after laparoscopic sleeve gastrectomy (LSG). **Methods:** This prospective observational cohort included patients who underwent LSG at a single academic health center between January 2017 and June 2022. We examined their body composition using InBody 720 or 770 and analyzed the factors associated with the percentage of total weight loss (%TWL) for 24 months. **Results:** The median body mass index (BMI) was 38.8 (interquartile range [IQR]: 35.6–46.7) preoperatively, 32.7 kg/m^2^ (IQR: 28.2–38.7) at 12 months postoperatively, and 33.9 kg/m^2^ (IQR: 29.1–40.1) at 24 months postoperatively. The lowest BMI was observed at 12 months (*p* < 0.001 vs. preoperative), followed by a significant increase at 24 months (*p* = 0.003). However, BMI remained significantly lower at 24 months than preoperatively (*p* < 0.001). The skeletal muscle mass to fat mass ratio (SMM/FM) was 0.59 (IQR: 0.50–0.71) preoperatively, 0.79 (IQR: 0.58–1.26) at 12 months, and 0.70 (IQR: 0.54–1.05) at 24 months, peaking at 12 months (*p* < 0.001 vs. preoperative) and decreasing significantly by 24 months (*p* < 0.001). Nevertheless, the SMM/FM ratio at 24 months remained higher than preoperative values (*p* < 0.001). Median body weight and %TWL were 86.0 kg and 15.6%, respectively, at 24 months after LSG. The SMM/FM ratio at 12 months was positively correlated with %TWL at 24 months after adjusting for age and sex. **Conclusions:** The effects of LSG persisted for up to 24 months postoperatively. The SMM/FM ratio 12 months after LSG was associated with the rate of weight loss at 24 months.

## 1. Introduction

Obesity is a chronic health problem that has reached epidemic prevalence worldwide [1,2,3,4]. It is associated with an increased risk of comorbidities such as type 2 diabetes mellitus (T2DM), dyslipidemia, hypertension, and sleep apnea syndrome. Obesity increases mortality and is, therefore, a serious global problem [5]. In Japan, the percentages of obese persons were 31.7% and 21% for males and females in 2022 [6]. Indications for bariatric surgery vary according to the opinions of various societies and insurance coverage but include severe obesity and treatment-resistant metabolic abnormalities due to obesity [7]. 892 bariatric surgeries were performed in 2023 [8]. Obesity treatments aim to restore the imbalance between energy intake and expenditure. However, conventional approaches such as lifestyle modification, dietary control, and increased physical activity are usually insufficient to achieve satisfactory weight loss [9,10]. Bariatric surgery has been demonstrated to be the most effective weight loss therapy available [11,12]. In Japan, laparoscopic sleeve gastrectomy (LSG) was the only bariatric procedure covered by the National Health Insurance until June 2024. Subsequently, LSG with duodenojejunal bypass began to be covered by the National Health Insurance, but LSG remains the primary procedure. LSG has been confirmed to be effective, and a nationwide survey conducted by the Japanese Survey of Morbid and Treatment-Resistant Obesity (a retrospective study) reported that the percentage of total weight loss (%TWL) at 12 months after LSG was 29.9% (322 Japanese patients underwent LSG) [12]. However, cases of insufficient weight loss or rebound were noted. Therefore, understanding the factors associated with negative outcomes is paramount for providing guidance in treatment selection and postoperative treatment strategy.

Many demographic and clinical factors, including age, sex, race, baseline weight, diabetes status, and duration, as well as environmental and psychosocial factors, have been associated with the degree of weight loss after metabolic/bariatric surgery [13,14,15,16,17,18,19,20,21,22,23]. In addition, body composition is considered important for postoperative weight loss (45 patients underwent bariatric surgery) [19]. However, no study has examined the relationship between postoperative body composition and weight loss in Japanese patients. We hypothesized that having more skeletal muscle and less body fat postoperatively would be associated with weight loss. Therefore, we aimed to examine the factors associated with the long-term maintenance of weight loss after LSG.

## 2. Materials and Methods

This single-center prospective cohort study was conducted at Ehime University Hospital and approved by the Ethics Committee of Ehime University Hospital (approval number: 1910003, on 28 October 2019). It included patients who underwent LSG at our hospital between January 2017 and June 2022 and were followed up for 24 months postoperatively until June 2024. Patients were selected in light of the insurance coverage available at the time: severely obese patients with a BMI ≥ 35 who did not respond adequately to medical therapy for at least 6 months and who had one or more of the following complications: diabetes, hypertension, dyslipidemia, or sleep apnea syndrome. After the LSG, nutritional guidance was provided monthly by a dietitian. Preoperative data were extracted within 2 weeks before LSG. Postoperative data were measured within 1 month before or after the scheduled measurement date (3, 6, 12, and 24 months after LSG). Body weight (BW) and composition, including muscle mass and body fat, were assessed using InBody 720 or 770 (InBody Japan Co., Ltd., Tokyo, Japan), direct segmental multifrequency bioelectrical impedance analyzer (DSM-BIA). InBody 720 and 770 use multi-frequency, a segmental measurement method, and an 8-point tactile electrode. The multi-frequency measurement is conducted by using multiple frequencies at 1, 5, 50, 250, 500, and 1000 kHz for each body segment (arms, trunk, and legs). This analyzer has been assessed in a variety of subjects, including normal populations and elderly patients, and closely correlates with the gold standard measurement by DEXA, underwater weight method, and air displacement plethysmography [24,25,26,27]. Also, this tool is not based on statistical data of any specific population. So, it is capable of accurately assessing people with very different physical types, whether obese or elderly [28,29]. Reliability is also ensured in severely obese patients [30,31]. Measurements were taken after participants had urinated, defecated, and rested for 5–10 min. To ensure accurate results, any exercise or activities that could induce sweating were prohibited before the DSM-BIA assessment. Twelve-hour, overnight-fasting, venous-blood samples were collected for assessing aspartate aminotransferase (AST), alanine aminotransferase (ALT), γ-glutamyl transpeptidase (GGT), total cholesterol (T-Chol), high-density lipoprotein cholesterol (HDL-Chol), low-density lipoprotein cholesterol (LDL-Chol), triglycerides (TG), C-reactive protein (CRP), uric acid (UA), creatinine (Cr), and hemoglobin A1c (HbA1c) levels. AST, ALT, and GGT were measured by enzyme assay corresponding to Japanese Society of Clinical Chemistry standardization. T-Chol, HDL-Chol, LDL-Chol, TG, and UA were measured by enzyme assay. CRP was measured by latex agglutination turbidimetric immunoassay. Cr was measured by chemical modification enzymatic assay. HbA1c was measured by high-performance liquid chromatography. Assessment variables included age, sex, BW (kg), body mass index (BMI) (kg/m^2^), fat mass (FM) (kg), percent body fat (%BF) (%), skeletal muscle mass (SMM) (kg), skeletal muscle percentage (SMP) (%), SMM to FM ratio (SMM/FM), skeletal muscle mass index (SMI) (kg/m^2^), and percentage of total weight loss (%TWL). SMI was computed by dividing the combined muscle mass of the upper and lower extremities by the individual’s height squared (kg/m^2^). %TWL was computed using this formula: (preoperative weight − postoperative weight)/preoperative weight × 100.

Regarding the effect of LSG, a %TWL of <20% at 24 months after LSG was defined as insufficient, whereas a %TWL of >20% was defined as sufficient [15]. First, the preoperative to 24-month postoperative change was analyzed. Second, we analyzed the characteristics associated with %TWL at 24 months after LSG. Third, to identify the characteristics related to rebound, the change in %TWL from 12 to 24 months (24-month %TWL–12-month %TWL) was analyzed. Data analysis and visualization were performed using EZR version 1.61 (Jichi Medical University Saitama Medical Center, Saitama, Japan) [32]. The results are expressed as medians with interquartile ranges for continuous variables. Baseline characteristics and body composition between 12 and 24 months after LSG were compared using the Mann–Whitney U test for continuous variables and Fisher’s exact test for categorical variables because it is not normally distributed. Changes in body composition were analyzed using the Wilcoxon signed-rank test. Characteristics associated with %TWL at 24 months and changes in %TWL from 12 to 24 months were analyzed using Spearman’s rank correlation coefficient and multiple regression analysis adjusted for age and sex. Statistical significance was set at *p* < 0.05.

## 3. Results

**Figure 1:**
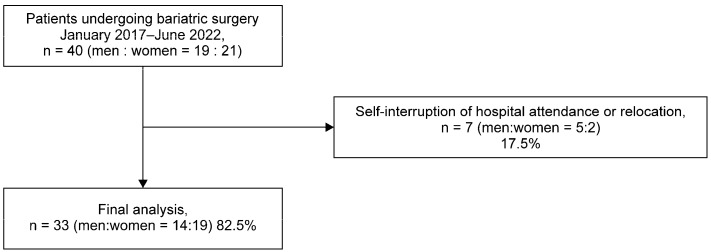
STROBE flowchart.

A total of 40 patients underwent surgery during the study period. Of these, seven (five men and two women) were excluded owing to self-interruption of hospital attendance or relocation. Data from the remaining 33 patients (14 men and 19 women) were analyzed (Figure 1).

### 3.1. Baseline Characteristics

The patients’ baseline characteristics are summarized in Table 1 and Table 2. The median age, BW, BMI, and %BF were 49 years, 105.3 kg, 38.8 kg/m^2^, and 48.9%, respectively. Men were younger than women; regarding their body composition, men had significantly higher BW (*p* < 0.001), SMM (*p* < 0.001), SMP (*p* < 0.001), SMM/FM (*p* < 0.001), and SMI (*p* < 0.001) but significantly lower %BF than women (*p* < 0.001). BMI, FM, and blood pressure were not significantly different between men and women. Blood biochemistry tests indicated higher UA and Cr levels and lower HDL-C levels in men than in women, with no significant differences in other parameters (Table 1). Patients in the sufficient group were younger than those in the insufficient group. The sufficient group had a higher percentage of men than the non-sufficient group. Regarding body composition, the sufficient group had higher SMM, SMP, SMI, and SMM/FM but lower %BF than the insufficient group, whereas BW, BMI, and FM were not significantly different. Blood pressure did not differ significantly between the two groups (*p* > 0.70). In the blood biochemistry tests, the sufficient group exhibited higher UA but lower HDL-Chol and HbA1c levels than the insufficient group, with no significant differences in other parameters (Table 2). There were 22 diabetic patients, 21 of whom had type 2 diabetes. 4 of these patients were using insulin, and 3 of them were able to discontinue insulin postoperatively. Both the one patient who could not discontinue insulin and the type 1 diabetic were able to reduce the number of insulin units.

### 3.2. Body Composition Change

**Figure 2:**
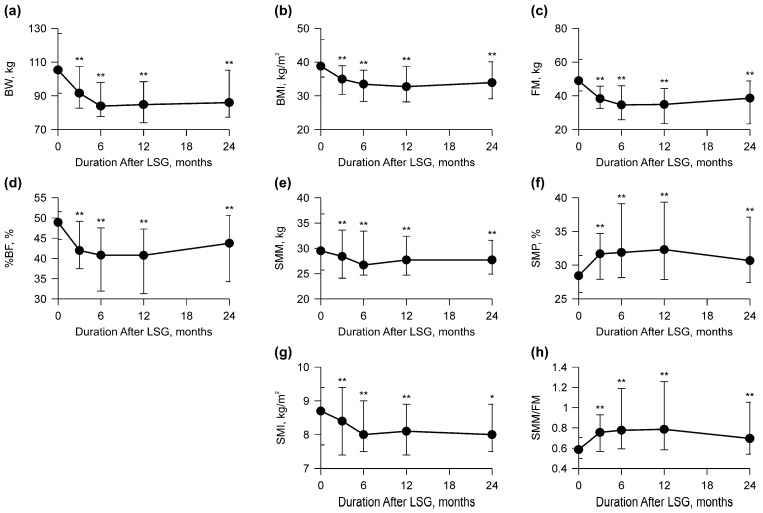
Graph of body composition before and 24 months after laparoscopic sleeve gastrectomy (LSG). The points in the graph represent medians with interquartile range values. (**a**) Body weight (BW) was the lowest after 6 months. (**b**) Body mass index (BMI) was the lowest after 12 months. (**c**) Fat mass (FM) was the lowest after 6 months. (**d**) Percent body fat (%BF) was the lowest at 6 and 12 months. (**e**) Skeletal muscle mass (SMM) was the lowest after 6 months. (**f**) Skeletal muscle percentage (SMP) was the highest after 12 months. (**g**) Skeletal muscle index (SMI) was the lowest after 6 and 24 months. (**h**) Skeletal muscle mass to fat mass ratio (SMM/FM) was the highest after 12 months. * *p* < 0.05, ** *p* < 0.001 vs. 0 months.

Changes in body composition, including BW, BMI, and muscle mass, are shown in Figure 2 and Table 3. BW, BMI, FM, and %BF significantly decreased within 12 months (*p* < 0.001) and significantly increased from 12 to 24 months after LSG (*p* < 0.004) (Figure 2a–d and Table 3). SMM and SMI significantly decreased at 6 months after LSG (*p* < 0.001) (Figure 2e and Table 3), whereas SMP and SMM/FM peaked at 12 months after LSG (*p* < 0.001) (Figure 2f,h and Table 3). There were no significant differences in SMM and SMI between 12 and 24 months (*p* > 0.41); however, SMP and SMM/FM decreased from 12 to 24 months after LSG (*p* < 0.001) (Figure 2e–h and Table 3). None of the parameters returned to preoperative values (*p* < 0.007) (Figure 2 and Table 3).

### 3.3. Distribution of %TWL at 24 Months After LSG

**Figure 3:**
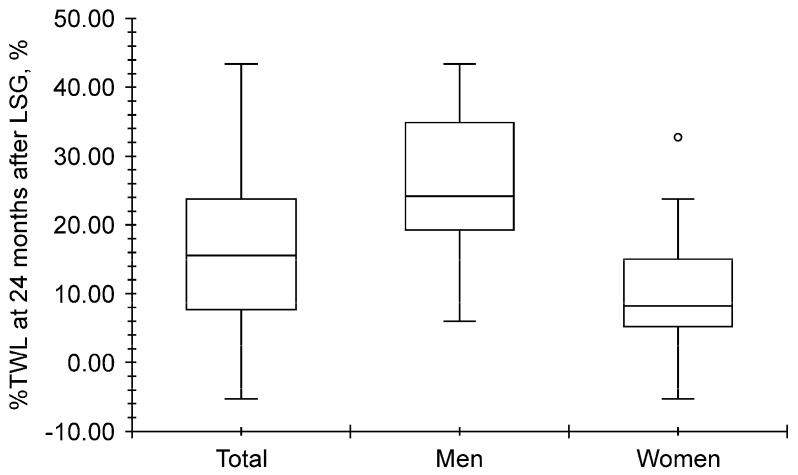
Distribution of the percentage of total weight loss (%TWL) at 24 months after laparoscopic sleeve gastrectomy (LSG). The 24-month-%TWL was greater in men than in women (open circle represents hazard value; *p* < 0.001).

As shown in Figure 3, the total median %TWL at 24 months after LSG was 15.6% (7.7–23.8%), and it was higher for men (24.2% [19.3–34.9%]) than for women (8.2% [5.2–15.0%], *p* < 0.001).

### 3.4. Factors Associated with %TWL at 24 Months After LSG

**Figure 4:**
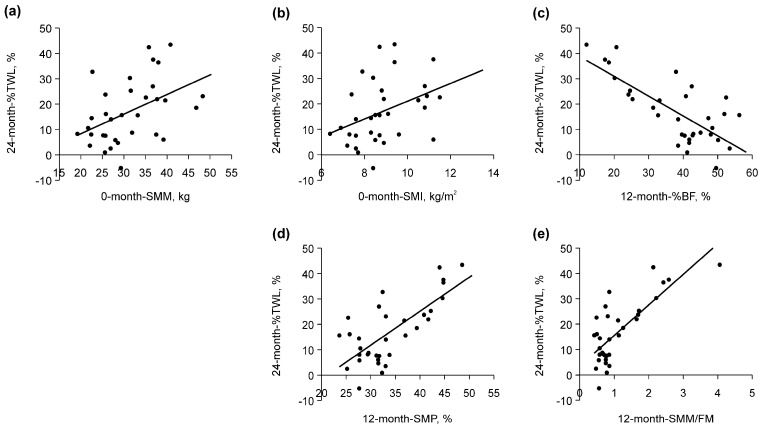
Correlation between the 24-month-percentage of total weight loss (%TWL) and each variable. (**a**) The zero-month skeletal muscle mass (SMM) exhibited a positive correlation with the %TWL at 24 months (r_s_ = 0.46, *p* = 0.007). (**b**) The zero-month skeletal muscle index (SMI) was positively correlated with the %TWL at 24 months (r_s_ = 0.41, *p* = 0.017). (**c**) The 12-month percent body fat (%BF) was negatively correlated with the %TWL at 24 months (r_s_ = −0.61, *p* < 0.001). (**d**) The 12-month skeletal muscle mass (SMP) exhibited a positive correlation with the %TWL at 24 months (r_s_ = 0.61, *p* < 0.001). (**e**) The 12-month SMM to FM ratio (SMM/FM) was positively correlated with the %TWL at 24 months (r_s_ = 0.61, *p* < 0.001).

The results of the univariate analysis of the variables are shown in Table 4. Among the preoperative variables, a positive correlation was observed between the %TWL 24 months after LSG, SMM, and SMI before LSG (Table 4 and Figure 4a,b). Among the postoperative variables, %BF, SMP, and SMM/FM at 12 months showed the strongest correlation with TWL% at 24 months after LSG (Table 4). A negative correlation was observed between %TWL 24 months after LSG and %BF (Table 4 and Figure 4c), and a positive correlation was observed between %TWL 24 months after LSG, SMP, and SMM/FM (Table 4 and Figure 4d,e).

Multiple regression analysis was performed using univariate analysis for factors that exhibited significance (Table 4). We selected SMM/FM at 12 months to assess skeletal muscle and body fat simultaneously. After adjusting for age and sex, these factors were found to be positively correlated (Table 5).

### 3.5. Change in %TWL from 12 to 24 Months After LSG

**Figure 5:**
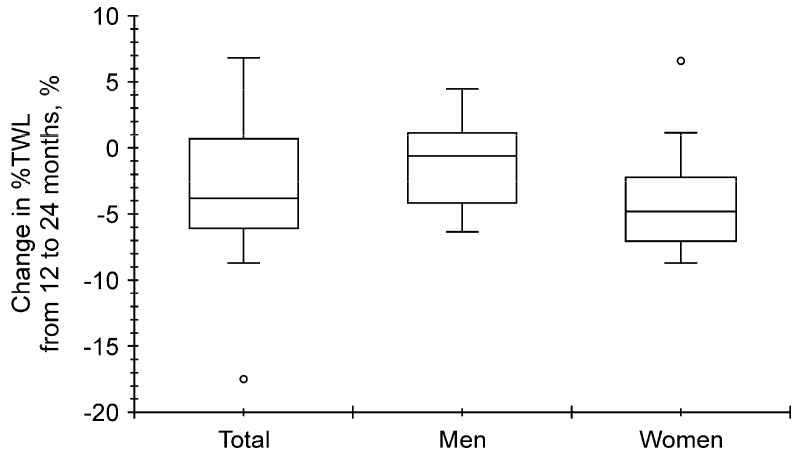
Change in the percentage of total weight loss (%TWL) from 12 to 24 months. No significant differences were observed between men and women (open circle represents hazard value; *p* = 0.051).

We next analyzed the change in %TWL from 12 to 24 months. The median change in %TWL from 12 to 24 months was −3.8% (−6.1–0.7%), −0.6% (−4.2–1.1%), and −4.8% (from −7.0% to −2.2%) for all patients, men, and women, respectively, and no significant differences were observed between men and women (*p* = 0.051) (Figure 5).

**Figure 6:**
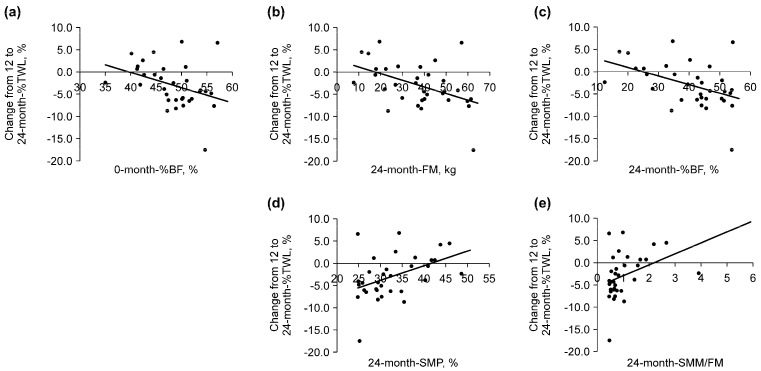
Correlation between the change in the percentage of total weight loss (%TWL) from 12 to 24 months and each variable. (**a**) The zero-month percent body fat (%BF) was negatively correlated with the change in the %TWL from 12 to 24 months (r_s_ = −0.44, *p* = 0.011). (**b**) The 24-month fat mass (FM) was negatively correlated with the change in the %TWL from 12 to 24 months (r_s_ = −0.47, *p* = 0.006). (**c**) The 24-month %BF was negatively correlated with the change in the %TWL from 12 to 24 months (r_s_ = −0.43, *p* = 0.012). (**d**) The 24-month skeletal muscle percentage (SMP) was positively correlated with the change in the %TWL from 12 to 24 months (r_s_ = 0.44, *p* = 0.010). (**e**) The 24-month skeletal muscle mass to fat mass ratio (SMM/FM) was positively correlated with the change in the %TWL from 12 to 24 months (r_s_ = 0.45, *p* = 0.008).

Table 6 displays the results of the correlation between the %TWL from 12 to 24 months and the evaluated variables. Among the preoperative variables, a negative correlation between the change in %TWL from 12 to 24 months after LSG and %BF before LSG (Table 6 and Figure 6a) and a positive correlation between the change in %TWL from 12 to 24 months after LSG and SMP, as well as SMM/FM (Table 6), were observed. Among the postoperative variables, a positive correlation was observed between the change in %TWL from 12 to 24 months after LSG and SMP as well as SMM/FM at 24 months after LSG (Table 6 and Figure 6d,e). In contrast, a negative correlation was observed between the change in %TWL from 12 to 24 months after LSG and BMI, FM, and %BF at 24 months (Table 6 and Figure 6b,c). Of these variables, baseline %BF, FM at 24 months, and %BF at 24 months exhibited stronger negative correlations, with correlation coefficients (r_s_) below −0.4. Conversely, skeletal muscle mass percentage (SMP) and the SMM/FM ratio at 24 months showed stronger positive correlations, with r_s_ values above 0.4 (Table 6, Figure 6).

From these variables, we selected SMM/FM, which reflects SMM and FM at 24 months after LSG. No significant differences were observed after adjusting for age and sex (*p* = 0.18). The correlation between the change in the %TWL 6–12 and 12–24 months after LSG was analyzed, but no correlation was found (*p* = 0.49).

The patients were divided into two groups according to whether they lost the most weight within 6 months and whether the LSG was sufficient. The changes in the %TWL are shown in Table 7. No significant differences were found (Table 7).

## 4. Discussion

This study examined the changes in weight and body composition after LSG and analyzed the factors related to the %TWL at 24 months after LSG. The most significant improvements in BW and body composition, specifically the greatest reduction in BMI and increase in the skeletal muscle mass to fat mass ratio (SMM/FM), were observed at 12 months after LSG. However, these benefits declined from 12 to 24 months, although BW at 24 months remained lower than preoperative levels. The median %TWL at 24 months was 15.6%. Notably, SMM/FM at 12 months was positively correlated with %TWL at 24 months (adjusted R-square = 0.58, estimate = 10.19, *p* < 0.001). These results suggest that high levels of SMM/FM at 12 months after LSG are important for maintaining weight loss after LSG. However, no factors associated with regaining weight from 12 to 24 months could be identified.

Several factors are reportedly associated with postoperative weight loss. Regarding sex, some studies indicate that women tend to lose more weight than men (e.g., data from 5057 laparoscopic Roux-en-Y gastric bypass [RYGB] and 2041 vertical sleeve gastrectomy [SG] patients) [33], whereas other reports indicate no significant differences between men and women (1012 patients undergoing either SG or RYGB) [15]. The impact of age on weight loss is also debated, with some studies reporting no association (750 patients undergoing SG or robotic-assisted RYGB) [20], whereas others suggest that younger patients may experience greater weight loss [15,21,22,23]. Additionally, higher preoperative BMI has been associated with greater weight loss in some studies (1012 patients undergoing either SG or RYGB) [15]. However, in our study, none of these factors were significantly linked to postoperative weight loss, possibly due to the limited sample size.

Several mechanisms may explain why the SMM/FM at 12 months postoperatively was associated with sustained weight loss at 24 months. Effective weight loss requires a caloric deficit, achieved by either increasing caloric expenditure or reducing caloric intake. Leptin, a hormone that suppresses appetite [34], is indirectly associated with BMI (as observed in a study of 87 lean and obese individuals) [35] and %BF (found in 136 normal-weight and 139 obese participants) [36]. However, obese patients often experience leptin resistance, reducing the hormone’s ability to curb appetite [36,37,38]. This suggests that relying solely on dietary interventions for weight loss can be challenging. While evidence from mouse models indicates that leptin might reduce the rewarding effects of exercise and thus lower activity levels [39], combining exercise with diet therapy has proven effective. Exercise promotes the secretion of N-lactoyl-phenylalanine, which helps inhibit feeding and prevent obesity [40]. Skeletal muscle plays a crucial role as a calorie-consuming organ, burning calories both during physical activity and at rest. Its basal metabolism is approximately 13 kcal/kg, compared to 4.5 kcal/kg for fat [41]. Higher basal metabolic rates have been associated with postoperative weight loss, as shown in a study involving 45 bariatric surgery patients [19]. Although muscle loss is a common concern during weight reduction, studies indicate that it can be preserved through exercise, particularly a combination of aerobic and resistance training, as demonstrated in 160 obese older adults [42].

After adjusting for age and sex, SMM/FM remained a significant factor for sustained weight loss, underscoring the role of combined diet and exercise, particularly a combination of aerobic and resistance training interventions in promoting long-term success after LSG. This suggests that early implementation of those regimens is crucial for all patients. However, no significant differences were found in preoperative and postoperative body composition, timing of the lowest BW, or changes in weight between 6 and 12 months post-LSG, which implies that adherence to adequate diet and exercise regimens may decline beyond the 12-month mark, making long-term maintenance challenging.

This study has some limitations. The sample size may have limited the ability to detect significant trends and associations between variables. Additionally, the absence of data on physical activity levels and nutritional intake precluded a detailed analysis of the relationship between body composition and postoperative diet and exercise regimens. Further research should examine how changes in these factors may mediate total weight loss (TWL) outcomes after LSG. The impact of comorbidities was not assessed in this study, leaving their influence on weight maintenance unclear. Moreover, while the effects of LSG persisted up to 24 months postoperatively, it remains uncertain how long these benefits can be sustained, as no specific factors associated with weight regain were identified. Future prospective studies are necessary to address these limitations and provide a clearer understanding of long-term outcomes.

## 5. Conclusions

The beneficial effects of LSG on weight loss were maintained up to 24 months postoperatively, with a median BW of 86.0 kg and a %TWL of 15.6%. No significant associations were found between weight loss and sex, age, or preoperative body composition. Importantly, the SMM/FM at 12 months was positively correlated with %TWL at 24 months.

## Figures and Tables

**Table 1:** Patients’ baseline characteristics.

Characteristics	Reference Range	Total	Men (n = 14)	Women (n = 19)	*p* Value
Age (years)	N/A	49 (40–53)	41.0 (33.0–49.5)	51.0 (48.5–54.5)	0.005
BW (kg)	N/A	105.3 (91.6–127.0)	120.5 (110.3–140.6)	95.6 (86.6–106.7)	<0.001
BMI (kg/m^2^)	18.5–25.0	38.8 (35.6–46.7)	41.1 (35.8–47.0)	38.8 (35.8–43.2)	0.62
FM (kg)	N/A	48.9 (42.9–61.6)	54.9 (45.7–63.6)	46.6 (42.6–56.8)	0.25
%BF (%)	N/A	48.9 (44.7–51.5)	44.6 (41.6–47.2)	50.8 (48.7–53.7)	<0.001
SMM (kg)	N/A	29.5 (25.7–36.8)	37.6 (36.0–39.5)	25.7 (22.6–38.3)	<0.001
SMP (%)	N/A	28.4 (26.0–31.4)	31.8 (29.2–33.0)	26.5 (25.1–28.3)	<0.001
SMI (kg/m^2^)	N/A	8.7 (7.7–9.4)	9.5 (8.8–10.8)	7.9 (7.5–8.5)	<0.001
SMM/FM	N/A	0.59 (0.50–0.41)	0.71 (0.63–0.79)	0.52 (0.47–0.58)	<0.001
SBP (mmHg)	<140	125.5 (120.0–140.8)	129.0 (119.5–140.3)	125.0 (120.0–141.5)	0.90
DBP (mmHg)	<90	76 (68–83)	74.0 (70.0–80.5)	76.5 (68.0–82.3)	0.70
AST (U/L)	9–37	28 (19–42)	25.0 (19.0–41.8)	28.0 (20.5–46.5)	0.58
ALT (U/L)	3–49	39 (22–72)	44 (23–70)	38 (22–65)	0.76
GGT (U/L)	6–71	38 (24–65)	36.5 (26.5–55.8)	40.0 (23.0–80.5)	0.76
T-Chol (mg/dL)	113–233	190 (164–208)	185.5 (166.8–216.3)	190.0 (164.0–203.5)	0.90
HDL-Chol (mg/dL)	50–60	46 (37–50)	38.5 (35.3–42.8)	48.0 (46.5–51.0)	0.002
LDL-Chol (mg/dL)	70–139	121 (98–132)	121.5 (100.5–139.5)	115.0 (96.0–128.5)	0.60
TG (mg/dL)	66–213	115 (86–143)	130.5 (106.0–152.5)	107.0 (76.5–139.5)	0.12
CRP (mg/dL)	0.00–0.20	0.42 (0.15–0.67)	0.46 (0.23–0.66)	0.42 (0.15–1.85)	0.90
UA (mg/dL)	2.7–5.8	6.0 (5.0–6.7)	7.0 (6.4–7.5)	5.1 (4.4–6.0)	<0.001
Cr (mg/dL)	0.46–0.79	0.68 (0.54–0.85)	0.89 (0.75–0.95)	0.58 (0.51–0.68)	<0.001
HbA1c (%)	4.6–6.2	6.3 (5.6–8.2)	6.0 (5.6–7.1)	7.3 (5.9–8.2)	0.22

BW, body weight; BMI, body mass index; FM, fat mass; %BF, percent body fat; SMM, skeletal muscle mass; SMP, skeletal muscle percentage; SMI, skeletal muscle index; SMM/FM, skeletal muscle mass to fat mass ratio; SBP, systolic blood pressure; DBP, diastolic blood pressure; AST, aspartate aminotransferase; ALT, alanine aminotransferase; GGT, γ-glutamyl transpeptidase; T-Chol, total cholesterol; HDL-Chol, high-density lipoprotein cholesterol; LDL-Chol, low-density lipoprotein cholesterol; TG, triglycerides; CRP, C-reactive protein; UA, uric acid; Cr, creatinine; HbA1c, hemoglobin A1c, N/A, not applicable.

**Table 2:** Baseline characteristics of the insufficient (≤19.9% body mass loss) and sufficient (> 20.0% body mass loss) groups.

Characteristics	Reference Range	Total (n = 33)	24 months—%TWL		
			≤19.9% (n = 20)	>20.0% (n = 13)	*p* Value
Age (years)	N/A	49 (40–53)	51 (44.5–55)	47 (33–50)	0.027
Sex, men/women	N/A	14/19	4/16	10/3	0.003
BW (kg)	N/A	105.3 (91.6–127.0)	100.5 (88.2–114.1)	113.9 (104.9–132.4)	0.077
BMI (kg/m^2^)	18.5–25.0	38.8 (35.6–46.7)	39.0 (35.9–45.0)	38.5 (35.3–47.0)	0.76
FM (kg)	N/A	48.9 (42.9–61.6)	49.0 (44.3–61.3)	48.9 (42.3–61.6)	0.93
%BF (%)	N/A	48.9 (44.7–51.5)	50.3 (48.1–52.4)	46.5 (42.4–47.4)	0.024
SMM (kg)	N/A	29.5 (25.7–36.8)	27.0 (24.5–30.1)	36.7 (31.6–38.0)	0.006
SMP (%)	N/A	28.4 (26.0–31.4)	27.4 (25.4–28.8)	30.1 (28.7–33.1)	0.011
SMI (kg/m^2^)	N/A	8.7 (7.7–9.4)	8.4 (7.6–8.8)	9.4 (8.7–10.8)	0.016
SMM/FM	N/A	0.59 (0.50–0.41)	0.54 (0.47–0.59)	0.65 (0.61–0.78)	0.016
SBP (mmHg)	<140	125.5 (120.0–140.8)	127.0 (119.8–142.3)	124.5 (120.3–137.8)	0.67
DBP (mmHg)	<90	76 (68–83)	76 (70–83)	73.0 (62.3–79.5)	0.29
AST (U/L)	9–37	28 (19–42)	28.0 (18.5–46.3)	26 (20–41)	0.78
ALT (U/L)	3–49	39 (22–72)	37.5 (22.0–76.5)	42 (23–64)	0.90
GGT (U/L)	6–71	38 (24–65)	40.5 (24.8–76.8)	33 (22–57)	0.73
T-Chol (mg/dL)	113–233	190 (164–208)	192.5 (174.0–208.0)	177 (164–226)	0.65
HDL-Chol (mg/dL)	50–60	46 (37–50)	48.0 (42.8–51.0)	39 (35–43)	0.007
LDL-Chol (mg/dL)	70–139	121 (98–132)	121.5 (98.8–129.0)	118 (95–143)	1.0
TG (mg/dL)	66–213	115 (86–143)	120 (82–145)	110 (97–143)	0.78
CRP (mg/dL)	0.00–0.20	0.42 (0.15–0.67)	0.56 (0.26–0.66)	0.30 (0.15–0.70)	0.40
UA (mg/dL)	2.7–5.8	6.0 (5.0–6.7)	5.7 (4.7–6.5)	6.5 (5.7–7.1)	0.046
Cr (mg/dL)	0.46–0.79	0.68 (0.54–0.85)	0.63 (0.53–0.72)	0.77 (0.68–0.93)	0.080
HbA1c (%)	4.6–6.2	6.3 (5.6–8.2)	7.4 (6.1–8.3)	5.8 (5.5–6.2)	0.014

%TWL, percentage of total weight loss; BW, body weight; BMI, body mass index; FM, fat mass; %BF, percent body fat; SMM, skeletal muscle mass; SMP, skeletal muscle percentage; SMI, skeletal muscle index; SMM/FM, skeletal muscle mass to fat mass ratio; SBP, systolic blood pressure; DBP, diastolic blood pressure; AST, aspartate aminotransferase; ALT, alanine aminotransferase; GGT, γ-glutamyl transpeptidase; T-Chol, total cholesterol; HDL-Chol, high-density lipoprotein cholesterol; LDL-Chol, low-density lipoprotein cholesterol; TG, triglycerides; CRP, C-reactive protein; UA, uric acid; Cr, creatinine; HbA1c, hemoglobin A1c, N/A, not applicable.

**Table 3:** Medians of body composition preoperatively to 24 months after surgery.

Body Composition	0 M	Time After Surgery			
		3 M	6 M	12 M	24 M
BW median (kg)	105.3	91.6	83.9	84.8	86.0
BW *p* value 0 M	-	<0.001	-	-	-
	-	-	<0.001	-	-
	-	-	-	<0.001	-
	-	-	-	-	<0.001
BW *p* value 3 M	-	-	<0.001	-	-
	-	-	-	<0.001	-
	-	-	-	-	0.19
BW *p* value 6 M	-	-	-	0.16	-
	-	-	-	-	0.19
BW *p* value 12 M	-	-	-	-	0.004
BMI median (kg/m^2^)	38.8	34.9	33.4	32.7	33.9
BMI *p* value 0 M	-	<0.001	-	-	-
	-	-	<0.001	-	-
	-	-	-	<0.001	-
	-	-	-	-	<0.001
BMI *p* value 3 M	-	-	<0.001	-	-
	-	-	-	0.002	-
	-	-	-	-	0.24
BMI *p* value 6 M	-	-	-	0.16	-
	-	-	-	-	0.17
BMI *p* value 12 M	-	-	-	-	0.003
FM median (kg)	48.9	38.3	34.6	34.9	38.6
FM *p* value 0 M	-	<0.001	-	-	-
	-	-	<0.001	-	-
	-	-	-	<0.001	-
	-	-	-	-	<0.001
FM *p* value 3 M	-	-	<0.001	-	-
	-	-	-	<0.001	-
	-	-	-	-	0.12
FM *p* value 6 M	-	-	-	0.15	-
	-	-	-	-	0.17
FM *p* value 12 M	-	-	-	-	<0.001
%BF median (%)	48.1	42.0	40.8	40.8	43.8
%BF *p* value 0 M	-	<0.001	-	-	-
	-	-	<0.001	-	-
	-	-	-	<0.001	-
	-	-	-	-	<0.001
%BF *p* value 3 M	-	-	<0.001	-	-
	-	-	-	<0.001	-
	-	-	-	-	0.088
%BF *p* value 6 M	-	-	-	0.20	-
	-	-	-	-	0.31
%BF *p* value 12 M	-	-	-	-	<0.001
SMM median (kg)	29.5	28.4	26.7	27.7	27.7
SMM *p* value 0 M	-	<0.001	-	-	-
	-	-	<0.001	-	-
	-	-	-	<0.001	-
	-	-	-	-	<0.001
SMM *p* value 3 M	-	-	0.87	-	-
	-	-	-	0.77	-
	-	-	-	-	0.86
SMM *p* value 6 M	-	-	-	0.48	-
	-	-	-	-	0.72
SMM *p* value 12 M	-	-	-	-	1.0
SMP median (%)	28.4	31.7	31.9	32.3	30.7
SMP *p* value 0 M	-	<0.001	-	-	-
	-	-	<0.001	-	-
	-	-	-	<0.001	-
	-	-	-	-	<0.001
SMP *p* value 3 M	-	-	<0.001	-	-
	-	-	-	<0.001	-
	-	-	-	-	0.024
SMP *p* value 6 M	-	-	-	0.16	-
	-	-	-	-	0.44
SMP *p* value 12 M	-	-	-	-	<0.001
SMI median (kg/m^2^)	8.7	8.4	8.0	8.1	8.0
SMI *p* value 0 M	-	<0.001	-	-	-
	-	-	<0.001	-	-
	-	-	-	<0.001	-
	-	-	-	-	0.007
SMI *p* value 3 M	-	-	0.59	-	-
	-	-	-	0.12	-
	-	-	-	-	0.43
SMI *p* value 6 M	-	-	-	0.092	-
	-	-	-	-	0.67
SMI *p* value 12 M	-	-	-	-	0.41
SMM/FM median	0.59	0.76	0.78	0.79	0.70
SMM/FM *p* value 0 M	-	<0.001	-	-	-
	-	-	<0.001	-	-
	-	-	-	<0.001	-
	-	-	-	-	<0.001
SMM/FM *p* value 3 M	-	-	<0.001	-	-
	-	-	-	<0.001	-
	-	-	-	-	0.017
SMM/FM *p* value 6 M	-	-	-	0.15	-
	-	-	-	-	0.28
SMM/FM *p* value 12 M	-	-	-	-	<0.001

BW, body weight; BMI, body mass index; FM, fat mass; %BF, percent body fat; SMM, skeletal muscle mass; SMP, skeletal muscle percentage; SMI, skeletal muscle index; SMM/FM, skeletal muscle mass to fat mass ratio.

**Table 4:** Variables associated with the percentage of total weight loss at 24 months after laparoscopic sleeve gastrectomy.

Time	Preoperatively		Time After LSG							
			3 Months		6 Months		12 Months		24 Months	
Variables	r_s_	*p*	r_s_	*p*	r_s_	*p*	r_s_	*p*	r_s_	*p*
Age (years)	−0.32	0.067	-	-	-	-	-	-	-	-
BW (kg)	0.32	0.073	0.10	0.59	−0.11	0.53	−0.31	0.084	−0.40	0.022
BMI (kg/m^2^)	0.11	0.55	−0.10	0.58	−0.27	0.12	−0.46	0.008	−0.52	0.002
FM (kg)	0.05	0.80	−0.20	0.27	−0.39	0.027	−0.56	<0.001	−0.55	0.001
%BF (%)	−0.33	0.061	−0.40	0.022	−0.42	0.015	−0.61	<0.001	−0.59	<0.001
SMM (kg)	0.46	0.007	0.35	0.044	0.33	0.062	0.27	0.12	0.23	0.21
SMP (%)	0.34	0.055	0.41	0.017	0.45	0.009	0.61	<0.001	0.60	<0.001
SMI (kg/m^2^)	0.41	0.017	0.29	0.10	0.21	0.25	0.12	0.51	−0.02	0.89
SMM/FM	0.33	0.062	0.40	0.023	0.44	0.012	0.61	<0.001	0.59	<0.001

r_s_, Spearman’s rank correlation coefficient; BW, body weight; BMI, body mass index; FM, fat mass; %BF, percent body fat; SMM, skeletal muscle mass; SMP, skeletal muscle percentage; SMI, skeletal muscle index; SMM/FM, skeletal muscle mass to fat mass ratio.

**Table 5:** Variables associated with the percentage of total weight loss 24 months after laparoscopic sleeve gastrectomy.

Explanatory Variable	Estimate	95% CI		SE	t Value	*p* Value
		Lower	Upper			
Age (years)	0.022	−0.35	0.40	0.18	0.12	0.90
Sex	5.19	−2.49	12.87	3.75	1.38	0.18
BMM/FM, 12 months after LSG	10.19	5.36	15.02	2.36	4.31	<0.001
Sample	33					
Adjusted R-square	0.58					
F-statistic	15.78					
Degree of freedom (3, 29)						

SE, standard error; CI, confidence interval; SMM/FM, skeletal muscle mass to fat mass ratio; LSG, laparoscopic sleeve gastrectomy.

**Table 6:** Variables associated with changes in the percentage of total weight loss from 12 to 24 months after laparoscopic sleeve gastrectomy.

Time	Preoperational		Time After LSG							
			3 Months		6 Months		12 Months		24 Months	
Variables	r_s_	*p*	r_s_	*p*	r_s_	*p*	r_s_	*p*	r_s_	*p*
Age (years)	−0.09	0.62	-	-	-	-	-	-	-	-
BW (kg)	0.02	0.93	0.003	0.96	−0.04	0.82	−0.08	0.65	−0.32	0.070
BMI (kg/m^2^)	−0.06	0.75	−0.09	0.63	−0.09	0.62	−0.17	0.34	−0.37	0.037
FM (kg)	−0.19	0.29	−0.21	0.25	−0.24	0.17	−0.27	0.13	−0.47	0.006
%BF (%)	−0.44	0.011	−0.27	0.13	−0.29	0.10	−0.29	0.097	−0.43	0.012
SMM (kg)	0.16	0.37	0.18	0.32	0.22	0.21	0.20	0.27	0.12	0.52
SMP (%)	0.36	0.039	0.29	0.099	0.32	0.071	0.29	0.10	0.44	0.010
SMI (kg/m^2^)	0.17	0.35	0.14	0.43	0.15	0.39	0.11	0.56	−0.09	0.61
SMM/FM	0.38	0.028	0.28	0.11	0.29	0.11	0.29	0.092	0.45	0.008

r_s_, Spearman’s rank correlation coefficient; BW, body weight; BMI, body mass index; FM, fat mass; %BF, percent body fat; SMM, skeletal muscle mass; SMP, skeletal muscle percentage; SMI, skeletal muscle index; SMM/FM, skeletal muscle mass to fat mass ratio.

**Table 7:** Comparison of the changes in the percentage of total weight loss from 12 to 24 months after laparoscopic sleeve gastrectomy between the two groups.

Minimum BW After Surgery	Number (Women)	Amount of Change in %TWL from 12 to 24 Months, %		
		Median	IQR	*p* Value
3 or 6 months	15 (10)	−2.8	(from −6.1 to −1.0)	0.94
12 months	18 (9)	−4.1	(−6.9–0.7)	
**%TWL at 12 M**	**Number (women)**	**Amount of change in %TWL from 12 to 24 months, %**		
		**Median**	**IQR**	***p* value**
Insufficient (≤19.9%)	17 (13)	−4.1	(from −6.0 to −1.4)	0.75
Sufficient (≥20.0%)	16 (6)	−3.1	(from −6.6 to −1.8)	
**%TWL at 24 M**	**Number (women)**	**Amount of change in %TWL from 12 to 24 months, %**		
		**Median**	**IQR**	***p* value**
Insufficient (≤19.9%)	20 (16)	−4.6	(from −6.1 to −1.8)	0.13
Sufficient (≥20.0%)	13 (3)	−0.6	(−4.3–2.6)	

BW, body weight; %TWL, percentage of total weight loss; IQR, interquartile range.

## Data Availability

The raw data supporting the conclusions of this article will be made available by the authors on request.
